# Spexin Promotes the Proliferation and Differentiation of C2C12 Cells In Vitro—The Effect of Exercise on SPX and SPX Receptor Expression in Skeletal Muscle In Vivo

**DOI:** 10.3390/genes13010081

**Published:** 2021-12-28

**Authors:** Natalia Leciejewska, Ewa Pruszyńska-Oszmałek, Karolina Mielnik, Maciej Głowacki, Tomasz P. Lehmann, Maciej Sassek, Bartosz Gawęda, Dawid Szczepankiewicz, Krzysztof W. Nowak, Paweł A. Kołodziejski

**Affiliations:** 1 Department of Animal Physiology, Biochemistry and Biostructure, Faculty of Veterinary Medicine and Animal Science, University of Life Sciences, 60-637 Poznan, Poland; natalia.leciejewska@up.poznan.pl (N.L.); ewa.pruszynska@up.poznan.pl (E.P.-O.); karo.mielnik@gmail.com (K.M.); maciej.sassek@up.poznan.pl (M.S.); dawid.szczepankiewicz@up.poznan.pl (D.S.); kwnowak@up.poznan.pl (K.W.N.); 2 Faculty of Physics, Adam Mickiewicz University in Poznan, 61-614 Poznan, Poland; 3 Department of Paediatric Orthopaedics and Traumatology, Faculty of Medicine, Poznan University of Medical Sciences, 61-701 Poznan, Poland; glowackimaciej@o2.pl; 4 Department of Biochemistry and Molecular Biology, Faculty of Medicine, Poznan University of Medical Sciences, 61-701 Poznan, Poland; tlehmann@ump.edu.pl; 5 Department of Preclinical Sciences and Infectious Diseases, Faculty of Veterinary Medicine and Animal Science, Poznań University of Life Sciences, 60-637 Poznan, Poland; bartosz.gaweda@up.poznan.pl

**Keywords:** spexin, C2C12 cells, skeletal muscle, differentiation

## Abstract

SPX (spexin) and its receptors GalR2 and GalR3 (galanin receptor subtype 2 and galanin receptor subtype 3) play an important role in the regulation of lipid and carbohydrate metabolism in human and animal fat tissue. However, little is still known about the role of this peptide in the metabolism of muscle. The aim of this study was to determine the impact of SPX on the metabolism, proliferation and differentiation of the skeletal muscle cell line C2C12. Moreover, we determined the effect of exercise on the SPX transduction pathway in mice skeletal muscle. We found that increased SPX, acting via GalR2 and GalR3 receptors, and ERK1/2 phosphorylation stimulated the proliferation of C2C12 cells (*p* < 0.01). We also noted that SPX stimulated the differentiation of C2C12 by increasing mRNA and protein levels of differentiation markers Myh, myogenin and MyoD (*p* < 0.01). SPX consequently promoted myoblast fusion into the myotubule (*p* < 0.01). Moreover, we found that, in the first stage (after 2 days) of myocyte differentiation, GalR2 and GalR3 were involved, whereas in the last stage (day six), the effect of SPX was mediated by the GalR3 isoform. We also noted that exercise stimulated SPX and GalR2 expression in mice skeletal muscle as well as an increase in SPX concentration in blood serum. These new insights may contribute to a better understanding of the role of SPX in the metabolism of skeletal muscle.

## 1. Introduction

In 2007, using bioinformatics techniques, a novel, highly conservative 14-amino-acid peptide was discovered [1]. This peptide was called spexin (SPX) or neuropeptide q (NPQ). The tissue expression of this peptide is very widespread in both human and animal organisms. Its presence has also been demonstrated in tissues involved in the regulation of carbohydrate–lipid metabolism, such as the liver, adipose tissue, pancreas, kidneys, muscle tissue and tissues of the gastrointestinal tract of the stomach in addition to the small and large intestines [2,3].

Many positive functions of this peptide have been demonstrated so far, especially in the case of its role in the context of the regulation of metabolic disorders related to diabetes and obesity [4,5]. However, most research has been focused on adipose tissue, the liver, the pancreas or the kidneys [6,7,8]. It has been shown, inter alia, that spexin is able to regulate lipogenesis and lipolysis processes in adipocytes [9], prevent inflammation in fat tissue [10], regulate insulin secretion [11], mitigate the metabolic changes and renal dysfunction accompanying obesity [12] and exhibit cardioprotective properties [13]. Despite the increasing knowledge on the role of SPX in the metabolism of metabolic disorders such as diabetes or obesity, there are still some pieces required to solve this puzzle. One of these main elements is the role of SPX in the metabolism of muscle cells, the function of which is extremely important in the aforementioned disorders and the development of the phenomenon of insulin resistance that accompanies them. Due to the fact that SPX is a pleiotropic peptide that activates two isoforms of the galanin receptor—GalR2 and GalR3—and thus many intracellular transmission pathways, e.g., ERK1/2, it can be suspected that it will also be important in the metabolism of skeletal muscles by influencing the processes of proliferation or differentiation [14,15,16].

Myogenesis is a fascinating process of growth and self-renewal in skeletal muscles. It is controlled by myogenic regulatory factors (MRFs) both prenatally and postnatally. Inactive satellite cells do not express MRFs. Their expression appears in an orderly manner during myogenesis. The first factor that rises in the early stages of activation and proliferation is Myf5, which causes the transition from progenitor cells into myoblasts [17]. Then, the expression of the MyoD factor is expressed. MyoD accumulates at the beginning of differentiation and starts the expression of the remaining MRFs. In the next step myoblasts further differentiate under the control of late MRFs: myogenin and Mrf4 [18]. In addition to regulatory factors myocytes begin to express essential muscle cell proteins, i.e., myosin heavy chains (MyHs) [19].

Therefore, using the markers demonstrated above we decided to investigate the role of SPX in the regulation of muscle cell tissue, such as in proliferation or differentiation. For this purpose, we used the murine cell line C2C12 for our in vitro study. Moreover, we demonstrated the effect of physical activity on SPX system expression in muscle tissue as well as the effect of training on metabolic markers and SPX concentration in blood serum in mice in vivo.

## 2. Materials and Methods

### 2.1. Cell Culture and the Induction of Differentiation

The C2C12 cell line was purchased from the European Collection of Authenticated Cell Cultures (ECACC). Cells were cultured in a growth medium (DMEM containing 4.5 g/L glucose and L-glutamine) supplemented with 10% fetal bovine serum (FBS) and 1% penicillin–streptomycin at 37 °C in a humidified 5% CO_2_ incubator. When cells reached 90% confluence the growth medium was replaced with a differentiation medium: DMEM containing 2% horse serum. During the experiments the medium was changed to a fresh one at two-day intervals.

### 2.2. Animals

C57BJ/6 mice (*n* = 14) were purchased from the Mossakowski Medical Research Centre Polish Academy of Sciences. The mice were housed under standard conditions (12/12 h light/dark cycle, 21 ± 1 °C). The study protocol was approved by the local Ethical Commission for Animal Care and Use in Poznan, Poland, University of Life Sciences (permission no. 39/2017). Mice were trained as we previously described [20]. In brief, animals (*n* = 7) were exposed to forced physical activity, that is, running on a horizontal rodent treadmill at 12 m/min for 30 min a day (5% grade/slope) for 30 days (Ugo Basile, cat no. 47300, Gemonio, Italy). The next seven animals constituted the control group, which was kept in standard laboratory conditions but not subjected to forced physical activity. After 30 days the animals were sacrificed by decapitation. Blood and tissues were placed at −80 °C until analysis. Before the analysis the quadriceps fragment was cleared of intramuscular adipose tissue using scissors and a magnifying glass, then placed in a tube with EXTRAzol (DNA, Gdansk, Poland) and homogenized using TissueLyser II (Qiagen, Germantown, MD, USA). RNA from muscle tissue was extracted according to the manufacturer’s instructions.

### 2.3. Blood Biochemical Analysis

Analyses of blood were performed using commercially available colorimetric assays from Pointe Scientific (Canton, MI, USA)—Glucose (Oxidase) (cat no. G7521), TG (cat no. T7532), Cholesterol (cat no. C7510), Albumin (cat no. A7502), Total Protein (cat no. T7528)—and an NEFA (NEFA-HR(2) Assay), Wako Chemicals (Neuss, Germany). Sample absorbances were read using a Synergy 2 multimode microplate reader (BioTek Instruments, Winooski, VT, USA).

The concentration of spexin in serum blood was determined using a commercially available Spexin RIA Kit (Phoenix Pharmaceuticals, Burlingame, CA, USA, cat no. RK-023-81). Quantifications of Gamma radiation were performed using a Wallac Wizard 1470 Gamma Counter (Perkin Elmer, Waltham, MA, USA).

### 2.4. Chemicals

Media and supplements were from Corning (Tewksbury, MA, USA). Spexin was purchased from Phoenix Pharmaceuticals. Galanin receptor antagonists were purchased from Tocris Bio-techne. Antibodies: anti-myogenin (sc-12732), anti-Myh (sc-376157) and anti-MyoD (sc-377460), anti-vinculin (sc-73614), anti-GALR2 (AF1463a), anti-GALR3 (AP16443), anti-phospho-ERK1/2 (sc-7383) and anti- ERK1/2 (sc-514302) were from Santa Cruz Biotechnology (Dallas, TX, USA) or Abgent (San Diego, CA, USA); anti-βactin A2228 and all secondary antibodies for Western blot were from Sigma-Aldrich (Taufkirchen, Germany). Unless otherwise stated all other reagents were from Sigma-Aldrich.

### 2.5. Cell Proliferation and Viability

C2C12 cells were seeded in 96-well plates in a growth medium and cultured for 24 h. The medium was subsequently removed and replaced with a serum-free medium supplemented with 0.2% BSA and treated with spexin in various concentrations for 24 h. After 24 h to investigate viability, cells were treated with an MTT solution (3-(4,5-dimethylthiazol-2-yl)-2,5-diphenyltetrazolium bromide) for 1 h. Then, the medium was removed, 100 μL of DMSO was added and the absorbance was detected by a Synergy 2 microplate reader (BioTek Instruments, Winooski, VT, USA).

### 2.6. ERK1/2 Phosphorylation and PCNA Analysis

C2C12 cells were seeded on six-well plates and cultured in a growth medium. Cells were subsequently washed with PBS and the medium was replaced with an experimental medium. Cells were incubated for 3 h. The medium was then changed once again for a medium supplemented with 1000 nM of spexin and cells were treated for 5, 10, 15, 30 and 45 min. For PCNA protein content analysis cells were also cultured on six-well plates with different concentrations of SPX (1, 10, 100 and 1000 nM) for 24 h in serum-free medium supplemented with 0.2% BSA. A medium supplemented with FBS was a positive control for the experiments.

After the experiments cells were lysed using a RIPA buffer, transferred to PCR tubes and centrifuged at 12,000× *g* for 15 min at 4 °C. The supernatant was transferred to new tubes, the protein concentration was determined using the BCA method and subsequently a Western blot was performed using 20 µg of total protein per line.

### 2.7. Quantitative RT-PCR

Total RNA was obtained with EXTRAzol Reagent (DNA Gdansk, Poland). In brief, to detach and lysate C2C12 cells EXTRAzol Reagent was added directly to the culture plate and the lysate was then transferred to an isolation PCR tube. In the case of RNA isolation from tissues, an additional step as described above was the mechanical homogenization of the samples. The isolation of RNA was conducted according to the manufacturer.

cDNA was synthesized from 1 μg of total RNA using a High-Capacity cDNA Reverse Transcription Kit (Life Technologies, Grand Island, NY, USA) according to the manufacturer’s instructions. Real-time PCR was performed using Quant Studio 12K Flex™ with 5 ×HOT FIREPol® Eva-Green® qPCR Mix Plus (ROX) and gene-specific primers. The specificity of the reaction products was tested by determining the melting points (0.1 C/s transition rate). Relative gene expression was evaluated by the delta delta CT (ΔΔCT) method with GAPDH as a reference gene. PCR primers are listed in Table 1:

### 2.8. Western Blotting

Total cellular proteins were extracted using a RIPA lysis buffer. Protein concentration was measured using a BCA protein concentration assay kit (Thermo Scientific, Waltham, MA, USA) according to the manufacturer’s instruction. Twenty micrograms of total proteins was separated by SDS-PAGE with 12% gel for most of the analyzed protein and 8% gel for MYH. Proteins were then transferred to PVDF membranes. The membranes were blocked with 3% BSA in TBS-Tween-20 (TBST) for 1 h at room temperature. Membranes were subsequently incubated overnight at 4 °C with primary antibodies (dilution of 1:1000). Then, membranes were washed with TBST and incubated with secondary antibodies (dilution of 1:5000) for 1 h. The signals were visualized using an enhanced chemiluminescent reagent and the expression of β-actin or vinculin was used for normalization. The band density was analyzed by TotalLab software.

### 2.9. Jenner-Giemsa Stain

Cells were seeded on six-well plates. When cells reached 90% confluence the growth medium was replaced with a differentiation medium: DMEM containing 2% horse serum supplemented with spexin in 100 nM and 1000 nM concentrations. After the 6th day of differentiation, the cells were fixed by methanol. The staining protocol was then performed as previously described [21]. In brief, the fusion index was calculated by staining the fixed cell and determining the number of nuclei in a myotubule compared to the total number of nuclei in the sample using an inverted Delta Optical IB-100 light microscope.

### 2.10. Statistical Analysis

Data were compared by using a one-way ANOVA followed by Dunnett’s post hoc test compared to untreated control cells (in the case of more than 2 groups) or by using an unpaired Student’s *t*-test (two-tailed distribution). If the data did not meet the assumptions of the *t*-test (Gaussian/normal distribution), a Mann–Whitney U test was used. Statistical significance was accepted at *p* < 0.05 (*) and *p* < 0.01 (**). All analyses were carried out in GraphPad Prism 6.0 software (GraphPad Software, San Diego, CA, USA).

## 3. Results

### 3.1. SPX, GalR2 and GalR3 Expression in C2C12 Cells

**Figure 1:**
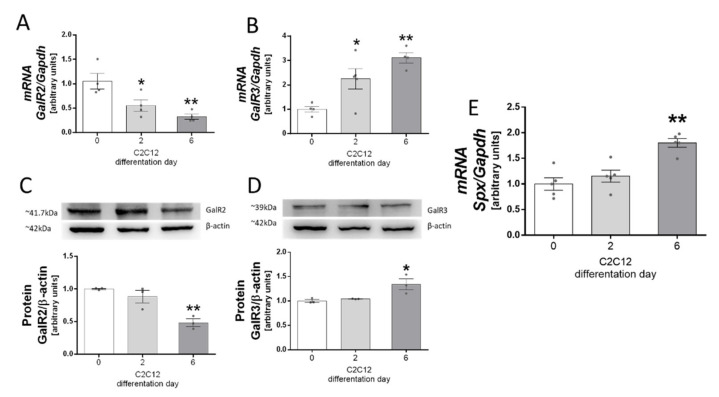
GalR2 and GalR3 mRNA (**A**,**B**) and protein (**C**,**D**) expression changes during the C2C12 differentiation process. SPX mRNA (**E**) levels in undifferentiated and differentiated cells. Values are presented as mean ± standard error of the mean (S.E.M.). Statistically significant differences are marked for *p* < 0.05 (*) and *p* < 0.01 (**) compared to day 0. The experiments were performed using *n* = 2 per investigated day. All experiments were duplicated.

We found that *GalR2* and *GalR3* mRNAs and proteins were expressed in C2C12 cells. Moreover, we found that GalR2 mRNA (*p* < 0.01) and protein expression (*p* < 0.01) decreased during the differentiation process (Figure 1A,C) in contrast to GalR3 mRNA (*p* < 0.01) and protein (*p* < 0.01) levels, which increased during this process (Figure 1B,D).

### 3.2. SPX Stimulates the Proliferation of C2C12 Cells

**Figure 2:**
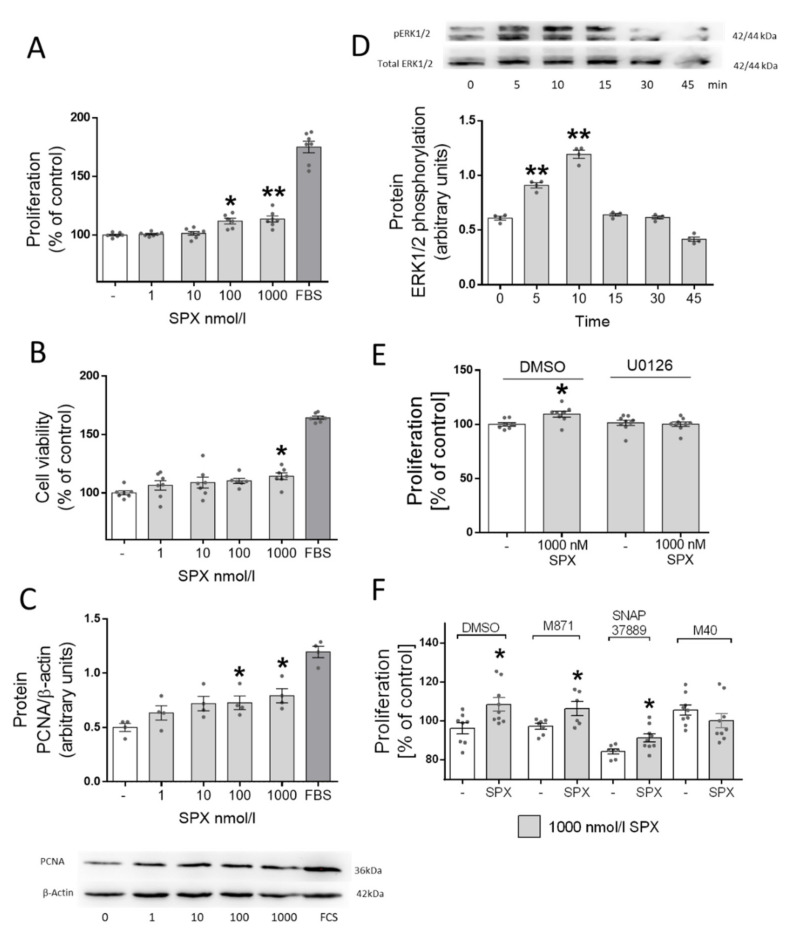
The effect of SPX on proliferation (**A**), cell viability (**B**), PCNA protein expression (**C**) and ERK1/2 phosphorylation (**D**). The effect of SPX on proliferation with or without ERK1/2 pharmacological blockers (**E**). The effect of different SPX on proliferation in the presence of different galanin receptor antagonists (**F**). Values are presented as mean ± standard error of the mean (S.E.M.) ((**A**,**B**,**E**,**F**) *n* = 6, 7; (**C**,**D**) *n* = 4). Statistically significant differences are marked for *p* < 0.05 (*) and *p* < 0.01 (**) compared to the corresponding control.

Next, we evaluated the effects of SPX on the differentiation process of C2C12 cells. We found that the addition of 100 (*p* < 0.05) and 1000 nM (*p* < 0.01) of SPX into the culture medium stimulated the proliferation process (Figure 2A, *p* < 0.01), whereas the addition of 1000 nM also slightly increased cell viability (Figure 2B, *p* < 0.05). We also found that the increase in proliferation was accompanied by an increase in proliferating cell nuclear antigen (PCNA) protein expression (Figure 2C, *p* < 0.05). PCNA expression is characteristic of proliferating cells; within skeletal muscle this is typical for activated satellite cells. Although MyoD is believed to be a key factor in initiating the transition from proliferation to differentiation, it has been shown that MyoD-expressing mononuclear myoblasts retain the ability to proliferate under a serum-rich medium; a marker of this process could be PCNA [22]. Previous research has demonstrated that SPX is able to activate ERK1/2 [23], and its activation is very often associated with the processes of cell proliferation and survival [24]. Therefore, we decided to investigate whether this effect could be mediated by ERK1/2. For this purpose, we checked whether the addition of SPX activates the phosphorylation of ERK1/2. We found a strong activation of ERK1/2 phosphorylation 5 and 10 min after the addition of 1000 nM of SPX into the incubation medium (Figure 2D, *p* < 0.01). To prove that the effect was mediated via ERK1/2 we performed an experiment with the pharmacological blocking of ERK1/2 by U0126. We noted that addition of this substance abolished the stimulatory effect of SPX on proliferation (Figure 2E, *p* < 0.01). In the last stage of this experiment we checked whether one or both of the galanin receptors were involved in the effects of SPX. Using 1000 nM of SPX, antagonists of GalR2 (M871) and GalR3 (SNAP37889) as well as a nonselective antagonist of M40 we proved that both isoforms of this receptor were involved in the effect of SPX on the proliferation process (Figure 2F, *p* < 0.05).

### 3.3. Effect of SPX on the Differentiation Process of C2C12 Cells

**Figure 3:**
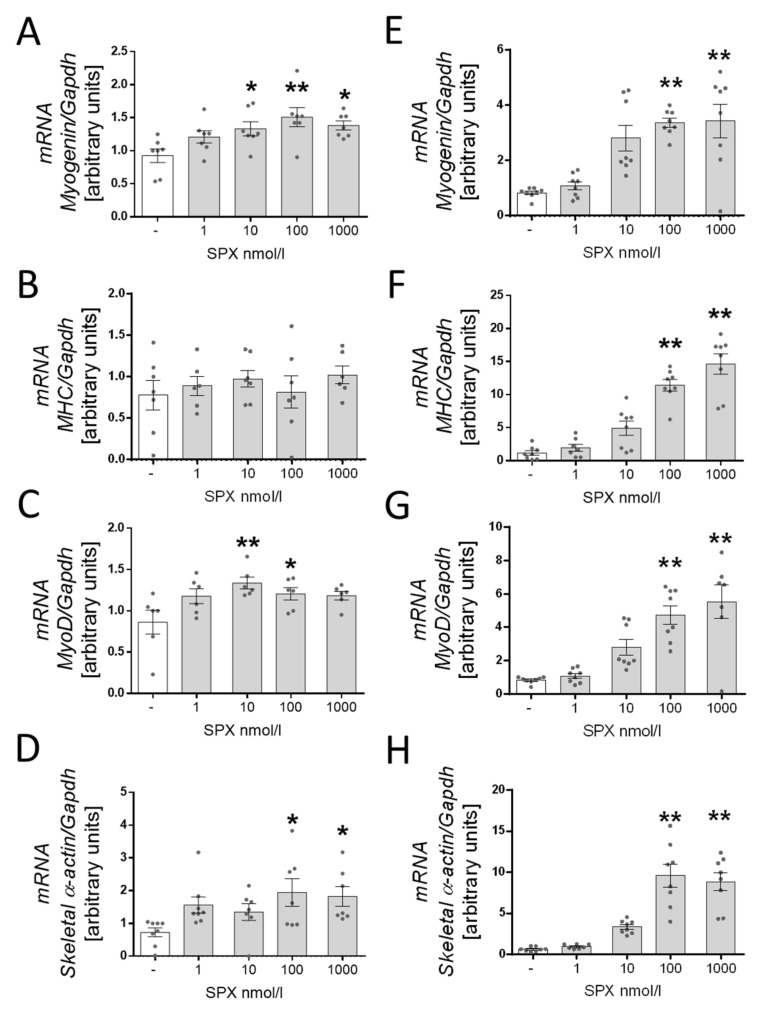
The effect of SPX on the mRNA expression of *myogenin* (**A**), *Myh4* (**B**), *MyoD* (**C**) and *skeletal α-actin* (**D**) after 2 days of differentiation and *myogenin* (**E**), *Myh4* (**F**), *MyoD* (**G**) and *skeletal α-actin* (**H**) after 6 days. Values are presented as mean ± standard error of the mean (S.E.M.) (*n* = 6, 8). Statistically significant differences are marked for *p* < 0.05 (*) and *p* < 0.01 (**) compared to the corresponding control.

**Figure 4:**
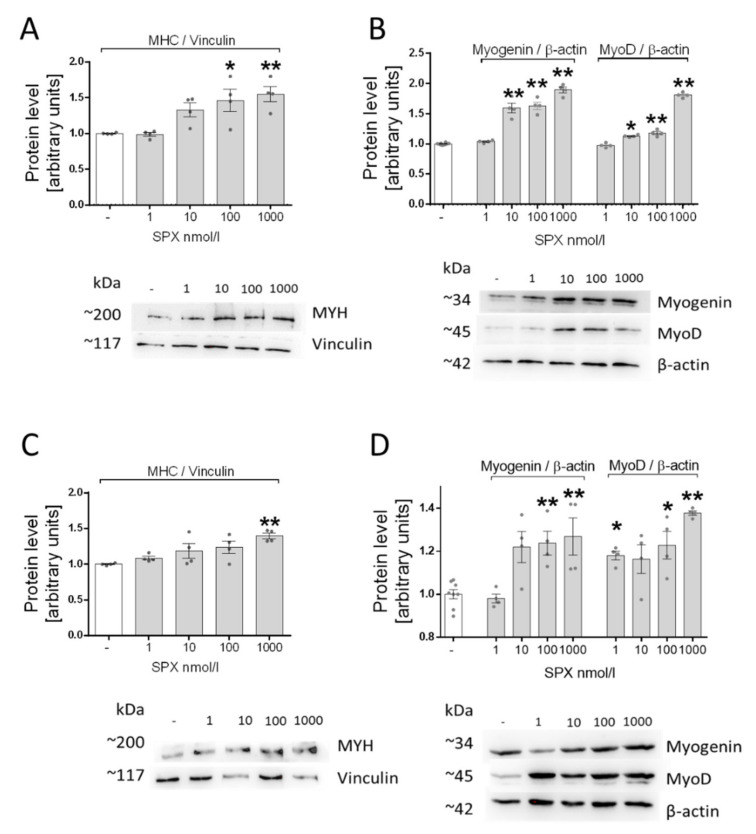
The effect of SPX on MYH (**A**), myogenin and MyoD (**B**) protein expression after 2 days and MYH (**C**), myogenin and MyoD (**D**) after 6 days. Values are presented as mean ± standard error of the mean (S.E.M.) (*n* = 4). Statistically significant differences are marked for *p* < 0.05 (*) and *p* < 0.01 (**) compared to the corresponding control.

Next, we decided to investigate the effect of SPX on differentiation markers in mRNA and protein levels after 2 and 6 days of initiating the differentiation process. We observed an increase in *myogenin* mRNA after the addition of 10 (*p* < 0.05), 100 (*p* < 0.01) and 1000 (*p* < 0.05) nM of SPX (Figure 3A) after 2 days as well as 100 (*p* < 0.01) and 1000 (*p* < 0.01) nM (Figure 3E) after 6 days. We also noted higher mRNA expression of *Myh4* on day 6 after the addition of 100 (*p* < 0.01) and 1000 (*p* < 0.01) nM of SPX (Figure 3F) and *MyoD* in groups with the addition of 10 (*p* < 0.05) and 100 *(p* < 0.01) nM of SPX on day 2 (Figure 3C) as well as on day 6 in groups with the addition of 100 (*p* < 0.05) and 1000 (*p* < 0.05) nM of SPX (Figure 3G). We also demonstrated that the addition of 100 and 1000 nM of SPX resulted in higher mRNA levels of the *skeletal α-actin* gene after 2 days (Figure 3D; *p* < 0.05) and 6 days of the differentiation process (Figure 3H; *p* < 0.01). The stimulating effect of SPX on the expression of differentiation markers was also confirmed in protein levels. We noted a higher protein level of Myh (Figure 4A; 100 nM, *p* < 0.05; 1000 nM, *p* < 0.01), myogenin (10 nM, *p* < 0.01; 100 nM, *p* < 0.01; 1000 nM, *p* < 0.01) and MyoD (10 nM, *p* < 0.05; 100 nM, *p* < 0.01; 1000 nM, *p* < 0.01) after 2 days (Figure 4B) and Myh (1000 nM, *p* < 0.01) (Figure 4C), myogenin (100 nM, *p* < 0.01; 1000 nM, *p* < 0.01) and MyoD (1 nM, *p* < 0.05; 100 nM, *p* < 0.05; 1000 nM, *p* < 0.01) after 6 days of differentiation (Figure 4D).

### 3.4. Fusion Index and the Identification of Receptors Involved in the Effect of SPX on the Differentiation Process

**Figure 5:**
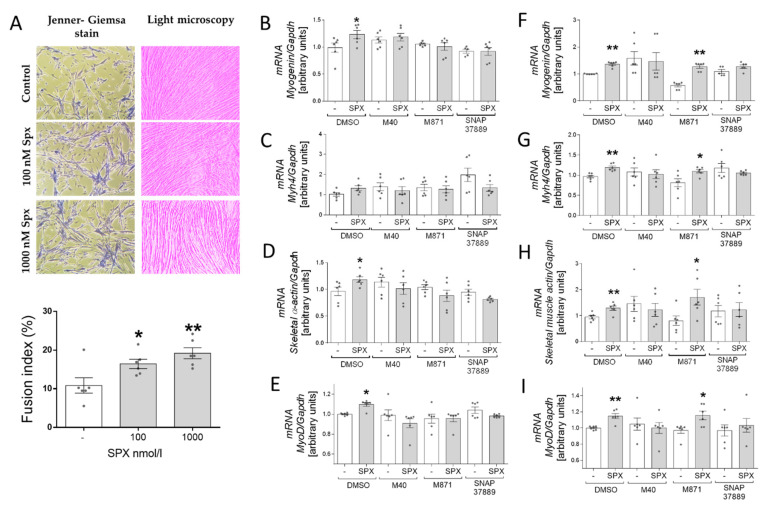
Fusion index changes after SPX treatment (**A**). The effect of SPX on *myogenin* (**B**), *Myh4* (**C**), *skeletal α-actin* (**D**) and *MyoD* (**E**) mRNA expression after 2 days of differentiation in the presence of the absence of galanin receptor antagonists (M40—nonselective GalRs antagonist; M871—selective GalR2 antagonist; and SNAP 37889—selective GalR3 antagonist) and *myogenin* (**F**), *Myh4* (**G**), *skeletal α-actin* (**H**) and *MyoD* (**I**) mRNA expression after 6 days of differentiation. Values are presented as mean ± standard error of the mean (S.E.M.) ((**A**), *n* = 6; (**B**–**I**), *n* = 5–6). Statistically significant differences are marked for *p* < 0.05 (*) and *p* < 0.01 (**) compared to the corresponding control.

Next, we decided to confirm the stimulating effect of SPX on the differentiation process of C2C12 cells by calculating the fusion index. We found that the addition of 100 and 1000 nM of SPX increases the fusion of nuclei into the myotubule compared to the total number of nuclei in the sample. We found that the addition of both doses of SPX increased the fusion index (*p* < 0.01) (Figure 4A). We also decided to identify the receptor involved in the effect of SPX on C2C12 differentiation. In this case we cultured cells with three different pharmacological antagonists of GalRs: SNAP 37889 (selective GalR3 antagonist); M871 (selective GalR2 antagonist) and M40 (nonselective galanin receptor antagonist) in the presence or absence of 100 nM of SPX. We observed increased mRNA expression of differentiation markers after 2 days of differentiation only in groups without antagonists (Figure 5B–E), which suggests that both receptors were involved in this process in the early stage. After 6 days of differentiation, we observed increased mRNA levels of *myogenin, Myh4, skeletal α-actin* and *myoD* genes in groups without any blocker as well as in groups with GalR2 antagonists (the addition of M781). These results suggest that in the late stage of the differentiation process the GalR3 receptor was involved and the role of GalR2 becomes limited.

### 3.5. Effect of Exercise on SPX Concentration and mRNA Expression of SPX, GalR2 and GalR3 in Skeletal Muscle in Mice

**Figure 6:**
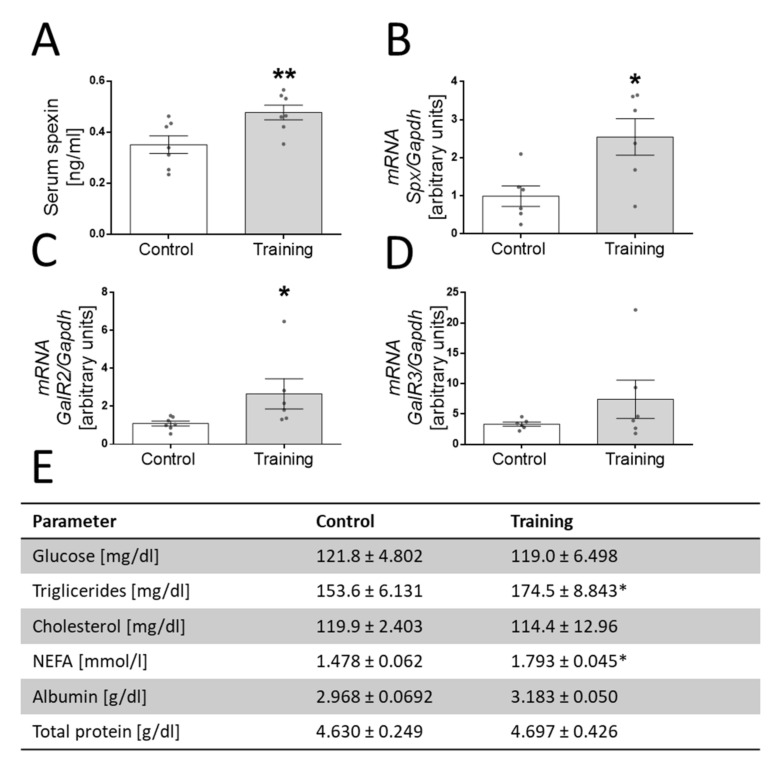
The effect of exercise on spexin concentration in blood serum in mice (**A**), *SPX* (**B**), *GalR2* (**C**) and *GalR3* (**D**) mRNA levels in skeletal muscle. The effect of exercise on basic biochemical parameters of blood in the mice subjected to the study (**E**) (*n* = 6, 7 per group). Statistically significant differences are marked for *p* < 0.05 (*) and *p* < 0.01 (**) compared to the control.

In the last part of our study, we decided to investigate the effect of exercise on SPX concentration in blood serum. Mice were trained on a horizontal rodent treadmill at 12 m/min for 30 min a day (5% grade/slope) for 30 days. We found higher blood SPX levels in mice after exercise (Figure 6A, *p* < 0.01). Moreover, we found that exercise increased the mRNA levels of SPX (Figure 6B, *p* < 0.05) and GalR2 (Figure 6C, *p* < 0.05), whereas we did not find an increase in the mRNA level of the GalR3 receptor (Figure 6D).

## 4. Discussion

In the present study we showed for the first time that SPX is able to regulate the proliferation, cell viability and differentiation of the C2C12 cell line. Moreover, we also showed that in the regulation of the proliferation process two isoforms/subtypes of galanin receptors are involved (GALR2 and GALR3), as is ERK1/2.

In the first part of our experiment we decided to investigate SPX mRNA and protein expression of *GalR2* and *GalR3* in C2C12 cells and skeletal muscle. Our results showed that both isoforms of galanin receptors were present in C2C12 cells and skeletal muscle in mice. These results confirmed previous discoveries which showed that *GalR2* and *GalR3* are present in C2C12 cells. Moreover, the changes in the expression of these proteins during the differentiation process are divergent. During the differentiation process we showed that the expression of *GalR2* decreased while the expression of *GalR3* increased. Many earlier studies have confirmed that SPX is widely expressed in many tissues of mammals, birds and other groups of animals [2,3,25,26], which indirectly suggests that SPX could play a role in the regulation of the metabolism of different tissues. Studies on the expression of GalR2 and GalR3 receptors in muscle tissue as well as in C2C12 cells have been more limited; however, both mRNA and protein studies indicate that both galanin receptor isoforms are expressed in muscle tissue. Additionally, mRNA and protein atlases confirm the expression of both isoforms of this receptor in the cells of muscle tissue, which was directly proven by our research and may confirm the role of the SPX/GALR2 and GALR3 axes in the metabolism of C2C12 cells.

The cell cycle is divided into several stages, which include the processes of proliferation and differentiation. Cell proliferation and differentiation show a remarkable inverse relationship, which is why we decided to investigate the effect of the addition of SPX addition on both processes [27]. Despite this, there are many biologically active substances that affect both processes equivalently (e.g., by the stimulation of both processes—differentiation and proliferation—as androgens do [28]) or antagonistically. Erythropoietin stimulates one of these processes (proliferation) and inhibits the differentiation of cells [29]. Due to the fact, as mentioned earlier, that the biological activity of SPX is regulated by two receptor isoforms, its effect on this process may also be binary. In investigating the effect of SPX on proliferation we detected that SPX slightly stimulates the proliferation process by affecting EKR1/2 kinase. Moreover, additional experiments also showed that this effect is mediated via both isoforms of GalRs and that the pharmacological blocking of this kinase results in the inhibition of this effect. It was previously shown that SPX is able to regulate the proliferation process; however, this effect was dependent on the type of tissue or cells on which the research was conducted. In our previous research performed on 3T3-L1 cells, we showed that SPX had no effect on this process [8]. Opposing results were obtained by us during studies using INS-1E β cells, whereas in the case of C2C12 cells we observed the stimulation of this process [11]. On the other hand, the research of Rucinski et al. proved that SPX could be considered as a proliferation inhibitor of adrenocortical cells [30]. These differences are probably due to the proportion of GalR2 and GalR3 receptors in particular tissues as well as the possibility of SPX activating both forms [30]. This seems to be even more interesting when considering the fact that the same group of scientists who conducted research into the effects of SPX on the proliferation of adrenocortical cells in their previous research did not show the expression of GalR2 or GalR3 in these cells [31], which would suggest that a different receptor is involved in the role of SPX in these cells. However, this can also be explained by genre differences; the study on SPX was conducted on rat cells whereas the study on the effects of galanin was conducted on human cells. Therefore, further studies are needed to elucidate the different role of SPX in the cell proliferation mechanism depending on the cell type.

However, based on the literature data and our results we can summarize that SPX is a peptide which is able to regulate the proliferation process in C2C12 cells via the activation of ERK1/2.

C2C12 cells, after reaching the appropriate degree of multiplication, begin the process of differentiation into mature cells/muscle fibers. Hence, we decided to check if SPX was also involved in differentiation. For this purpose, we decided to study the expression of mRNA and of Myh, myogenin and MyoD proteins, the basic differentiation markers; we found that SPX was able to stimulate differentiation. In 2010 Porzionato et al. demonstrated that SPX is widely expressed in rat tissues, particularly in gastrointestinal epithelial cells, which are considered to be rapidly proliferating and differentiating cells; this may suggest that SPX is involved in cell differentiation [2]. Our previous research on the role of SPX in adipose tissue differentiation demonstrated that this peptide is able to inhibit differentiation in 3T3-L1 cells [8]. We thought that SPX might affect the differentiation of muscle cells due to many similar metabolism features of 3T3-L1 and C2C12 myoblasts. It has been indicated in the literature concerning SPX that SPX is involved in the regulation of fat and carbohydrate metabolism in many peripheral tissues [4,7,32,33]; therefore, we assumed that the metabolism of skeletal muscle, as one of the largest consumers of both carbohydrates and lipids, could be regulated by SPX. We found that SPX stimulated the mRNA and protein expression of differentiation markers, which confirmed that this peptide accelerates the differentiation process of C2C12 myoblasts. Surprisingly, our research confirmed that the effects of spexin on differentiation are mediated by the GalR3 receptor. By using blockers of both GalR isoforms we proved that the effect of SPX was only abolished after using GalR3 blockers (SNAP 37889).

This effect of SPX was confirmed by Jenner–Giemsa staining, which also confirmed that SPX stimulates the formation of myotubes. Despite the fact that we showed that the GalR3 receptor appeared to be more important for the myoblast differentiation, data in the literature also indicate the significance of GalR2 and SPX in this process. In 2018 it was proven that the activation of GalR2 attenuates insulin resistance in the skeletal muscle of obese mice [15]. This suggests that this form of receptor and possible SPX effect via this receptor focuses on the regulation by insulin; however, this thesis requires further research. Moreover, the relationship between the number of different isoforms of galanin receptors seems to be very important. In the initial stage (undifferentiated cells), when GalR2 expression is high, SPX stimulates the proliferation process and then begins to exert its influence on differentiation through the GalR3 receptor, the expression of which increases with differentiation. GalR2 is a receptor that is widely expressed in many peripheral tissues, such as the gastrointestinal tract, pancreas, kidneys, muscles, adipose tissue and many others [34,35]. Many intracellular signaling pathways are regulated by G proteins coupled with this receptor subtype. The activation of GalR2 via Gq/11 stimulates phospholipase C (PLC), which results in the mobilization of Ca^2+^ and the activation of protein kinase C (PKC); via G0 activates the mitogen-activated protein kinase (MAPK) pathway; and via Giα proteins inhibits adenylyl cyclase (AC) [36], and these pathways largely participate in the proliferative activity of the cells, which include the aforementioned MAPK pathway. In contrast, GalR3 receptor activation is associated with the inhibition of the AC pathway with concomitant membrane hyperpolarization [37], which, as previous studies have shown, triggers myogenin and expression during myoblast differentiation [38]. Our studies have also shown that the expression of GalR2 was higher at the beginning of the experiment (in undifferentiated cells), where they have the highest proliferative potential, and decreased during differentiation, while the expression of GalR3 increased, which indirectly confirms our results about the effect of SPX on the proliferation and differentiation of cells. However, further research is required to fully confirm this thesis.

Our research was also inspired by the results of an in vivo experiment which showed that the 30-day administration of SPX in obese mice increased the levels of lean tissue in these animals, which also suggested that SPX is able to increase muscle mass [32]. However, what is interesting is that this effect was only observed in obese mice, not in those that were healthy or diabetic. This led us to considering the possibility of spexin being indirectly involved in myogenesis. Having material collected from animals from another project in which animals were subjected to physical exercise, we asked ourselves whether physical exercise could affect the expression of SPX. We were also prompted by the publications of Khadir et al. and Mohammadi et al., in which the authors showed that SPX can be a good marker of the effect of exercise on obese and diabetic people [39,40]. We noted that exercise increased the concentration of SPX in mice and increased the SPX mRNA level in skeletal muscle in mice, which also indirectly confirmed that SPX could be a regulator of skeletal muscle metabolism in vivo. An additional aspect that has been described and that may be relevant is the demonstration of an increase in GalR2 expression, induced by exercise, in mice. This fact, in connection with the role of this receptor in the proliferation of muscle cells, may indirectly indicate the beneficial effect of exercise in this tissue and enhance the regenerative properties of muscle cells after exercise.

Although this is the first research on the effects of SPX on the metabolism of skeletal muscle tissue, we are aware that it is very preliminary and requires further development to fully describe the role of SPX in the metabolism of this tissue. Our study has several limitations, one of which is the lack of the identification of intracellular pathways activated by SPX during the differentiation process. We also did not investigate the effect of SPX on muscle differentiation in vivo.

In summary, we demonstrated that SPX, GalR2 and GalR3 are present in C2C12 cells on the mRNA and protein levels; moreover, we also showed that SPX slightly stimulates the proliferation process via both isoforms of galanin receptors and by the activation of EKR1/2. We also proved that SPX is involved in the differentiation process of C2C12 cells, and that exercise stimulates its expression, leading to an increased concentration of SPX in blood serum and its expression in skeletal muscle cells. Overall, these results show that SPX increased in trained mice (in vivo) and directly modulated the metabolism of skeletal muscle cells in vitro via GalR2 and GalR3.

## Figures and Tables

**Table 1:** Primer sequence used for real-time PCR.

Target	Forward Primer (5′ > 3′)	Reverse Primer (5′ > 3′)	Product (bp)
*Myogenin*	CGGTGGAGGATATGTCTGTTG	GGTGTTAGCCTTATGTGAATGG	215
*MyoD*	AGCACTACAGTGGCGACTCA	GGCCGCTGTAATCCATCAT	75
*Skeletal α-actin*	CAGAGCAAGCGAGGTATCC	GTCCCCAGAATCCAACACG	297
*Myh4*	CTTGCGGTCCTCCTCGGTCTGGT	CGCCCACCTGGAGCGGATGA	250
*Spexin*	TCCTTCTCCTGGTGCTGTCT	TCTGGGTTTCGTCTTTCTGG	187
*GalR2*	CTTAAAGGCGCCCCATGT	CACTAGCGAGTCACACTGTTCC	72
*GalR3*	CGGCCGTCTCAGTGGATA	CGGCCGTCTCAGTGGATA	131
*Gapdh*	ATGGTGAAGGTCGGTGTGA	AATCTCCACTTTGCCACTGC	84

## Data Availability

The data presented in this study are available on reasonable request from the corresponding author.
